# Functional and molecular neuroimaging of menopause and hormone replacement therapy

**DOI:** 10.3389/fnins.2014.00388

**Published:** 2014-12-08

**Authors:** Erika Comasco, Vibe G. Frokjaer, Inger Sundström-Poromaa

**Affiliations:** ^1^Department of Neuroscience, Uppsala UniversityUppsala, Sweden; ^2^Department of Women's and Children's Health, Uppsala UniversityUppsala, Sweden; ^3^Department of Neurology, Center for Integrated Molecular Brain Imaging and Neurobiology Research Unit 6931, Copenhagen University HospitalCopenhagen, Denmark

**Keywords:** estrogens, fMRI, HRT, hormones, menopause, neuroimaging, PET, SPECT

## Abstract

The level of gonadal hormones to which the female brain is exposed considerably changes across the menopausal transition, which in turn, is likely to be of great relevance for neurodegenerative diseases and psychiatric disorders. However, the neurobiological consequences of these hormone fluctuations and of hormone replacement therapy in the menopause have only begun to be understood. The present review summarizes the findings of thirty-five studies of human brain function, including functional magnetic resonance imaging, positron and single-photon computed emission tomography studies, in peri- and postmenopausal women treated with estrogen, or estrogen-progestagen replacement therapy. Seven studies using gonadotropin-releasing hormone agonist intervention as a model of hormonal withdrawal are also included. Cognitive paradigms are employed by the majority of studies evaluating the effect of unopposed estrogen or estrogen-progestagen treatment on peri- and postmenopausal women's brain. In randomized-controlled trials, estrogen treatment enhances activation of fronto-cingulate regions during cognitive functioning, though in many cases no difference in cognitive performance was present. Progestagens seems to counteract the effects of estrogens. Findings on cognitive functioning during acute ovarian hormone withdrawal suggest a decrease in activation of the left inferior frontal gyrus, thus essentially corroborating the findings in postmenopausal women. Studies of the cholinergic and serotonergic systems indicate these systems as biological mediators of hormonal influences on the brain. More, hormonal replacement appears to increase cerebral blood flow in several cortical regions. On the other hand, studies on emotion processing in postmenopausal women are lacking. These results call for well-powered randomized-controlled multi-modal prospective neuroimaging studies as well as investigation on the related molecular mechanisms of effects of menopausal hormonal variations on the brain.

## Background

The change in ovarian hormone levels to which the female brain is exposed during the menopausal transition is likely relevant for neurodegenerative diseases as well as psychiatric disorders. However, the effects of the peri- and postmenopausal estradiol level decline and hormone replacement therapy (HRT) on the brain have only begun to be understood (Mueller et al., 2014). Undeniably, a substantial lack of agreement on the beneficial vs. detrimental effect of HRT on mental health, and concerns about potential adverse effects exist (Henderson and Greicius, 2010). Thus, the prevention and clinical management of mental health problems during the menopausal transition suffers from uncertainty, which renders research in this field of outmost importance.

During the last two decades menopause researchers have opted for an intermediate phenotype-based approach to further the psychobiological dissection of menopausal hormonal transition and HRT effects on mental health. Indeed, intermediate phenotypes (e.g., biochemical, endocrine, anatomical, physiological) represent quantifiable basic biological or behavioral processes, which are expected to be closer to the genetic underpinning as well as to the psychological functions that might be affected (Meyer-Lindenberg and Weinberger, 2006; Rasetti and Weinberger, 2011). Neuroimaging techniques are certainly suitable tools for investigating potential intermediate phenotypes related to the neurobiological correlates of menopause and HRT.

The present review summarizes the findings of studies of human brain function (i.e., functional magnetic resonance imaging (fMRI), positron emission tomography (PET), single-photon emission computed tomography (SPECT/SPET), and magnetic resonance spectroscopy (MRS), of perimenopausal and postmenopausal women treated with unopposed estrogen or combined estrogen-progestagen replacement therapy. Additionally, studies of gonadotropin-releasing hormone (GnRH) agonist treatment, as a pharmacological model of menopause, are also considered.

## State-of-the-art

To date, 35 functional neuroimaging studies have investigated brain function in peri- and postmenopausal women, and seven additional studies have used GnRH agonist intervention as a model of menopause (Tables 1–**6**). However, all studies are statistically under-powered since they include small samples, and they are also characterized by high heterogeneity in terms of study design. Differences regarding the study designs include: controlled randomization vs. cross-sectional trials, parallel vs. cross-over design, baseline vs. placebo control state, duration of the HRT use, unopposed estradiol vs. combined estrogen-progestagen therapy, route of administration and posology/dose of HRT, time between menopause and start of HRT (“critical window”), and chronological and reproductive age of the women. Dissimilarities regarding the neuroimaging data regard: neuroimaging techniques, neuropsychological paradigms within a cognitive domain, and statistical approaches for neuroimaging data processing and analysis. All together, these factors impeded any meta-analytical statistics.

**Table 1:** **Randomized controlled trial longitudinal studies neuroimaging cognitive functioning during HRT**.

**Study**	**Subjects [STRAW stage]**	**Age (y)**	**Hormonal treatment**	**Technique**	**Test**	**Comparison**	**Results**	**Notes**
**E**								
Dumas et al., 2010	20	60 ± 6	gr1 (*n* = 10): E(3m)	fMRI	Visual verbal working memory	Placebo vs. ET	ET: [3 Back] ↑ miFG (BA 8), sFG l (BA 10+8), iFG r (BA 47), miTG r (BA 21), CG l (BA 24), precG l (BA 4), postcG l (BA 4), pCun r (BA 31);	= Accuracy, but variable bias in performance
	[S+2]	60 ± 5	gr2 (*n* = 10): placebo (3m) (pastET, *n* = 6)	(2x)			ET: [2 Back] ↑ mFG r (BA 8), iFG r (BA 47), miFG r (BA 8), postcG l (BA 4);	
							NT: [1 Back] ↑ Ins r (BA 13), precG l (BA 6); ET: [0 Back] ↑ miTG r (BA 21), sTG r (BA 41)	
Joffe et al., 2006	11	51 ± 3	PeriMP	fMRI	Verbal working memory	Placebo vs. ET	ET: ↑ mFL, FG (BA11), PCL/FL (BA5), pcG/PL(BA1), PL, Cau r, Thal r	= Performance, but HT: less errors of perseveration in verbal recall
	[−2≤S≤+1C]	51 ± 4	gr1 (*n* = 5): baseline + transdermal E(12w)	(2x)	Spatial working memory	Placebo vs. ET	ET: [5–3 item] ↑ CG (BA32), sFG (BA9), Cun, pC (BA29)	
			gr2 (*n* = 6): baseline + placebo (12w)					
Shaywitz et al., 1999	46	51 ± 5	Pre- to postMP	fMRI	Verbal and non-verbal working memory	Placebo vs. ET	ET: [storage, verbal memory] ↑iPL, sFG [retrieval] ↑sFG r	= Performance
	[S:+1A]	(33–61)	gr1: CEE (3w) + wash-out (2w) and placebo (3w)	(2x)			ET: HERA effect: ↑ l vs. r during encoding, ↑ r vs. l during retrieval	
			gr2: placebo (3w) + wash-out (2w) and CEE (3w)					
**E+P**								
Davison et al., 2013	13	53	PostMP	fMRI	Verbal fluency	Baseline vs. HT	n.s	= Performance
	[S:≥+1A]	(50–54)	gr1 (*n* = 6): baseline + E P (6m)	(2x)	Mental rotation	placebo vs. HT		
		53	gr2 (*n* = 7): baseline + placebo (6m)					
		(50–55)						
Persad et al., 2009	10	60 ± 1	PostMP	fMRI	Verbal memory	Placebo vs. HT	HT: ↑ pFC l (BA6+9), daC/mFC (BA24+32), pC (BA6), PC l (BA40+39)	= Performance
	[S:≥+1C]	(56–60)	gr1: E P (4w) + wash-out (4w) and placebo (4w)	(2x)				
			gr2: placebo (4w) + wash-out (4w) and E P (4w)					
Smith et al., 2006	10	57 ± 1	PostMP	fMRI	Visual working memory	Placebo vs. HT	HT: ↑ pFC (BA44+45)	= Performance
	[S:≥+1C]	(50–60)	gr1: E P (4w) + wash-out (4w) and placebo (4w)	(2x)				
			gr2: placebo (4w) + wash-out (4w) and E P (4w)					

See Appendix for acronyms/abbreviations.

### Cognitive functioning

The great majority of studies evaluating the effect of unopposed estrogen or combined estrogen-progestagen treatment on the menopausal and postmenopausal brain used cognitive paradigms (Tables 1, 2). Longitudinal, randomized controlled trial studies of the effects of estrogen treatment—three to twelve weeks in duration—indicated a higher activation of fronto-cingulate regions during cognitive functioning (Table 1), as assessed by fMRI using verbal, non-verbal and spatial working memory tasks. However, in spite of higher activation in fronto-cingulate regions, no differences in working memory performance were noted (Shaywitz et al., 1999; Joffe et al., 2006; Dumas et al., 2010). One sample of healthy postmenopausal women with an average of 7 years of prior estrogen use, and randomly assigned to a placebo or three-month hormonal treatment, showed an estradiol, memory load-dependent, effect on frontal activation, with a greater modulatory effect during the more difficult visual verbal working memory task (Dumas et al., 2010). During a verbal memory task, inferior frontal and posterior parietal regions, and during a spatial working memory task, frontal, posterior cingulate, and parietal regions, activation was greater in peri- and post-menopausal women receiving a three-week transdermal estradiol treatment compared to placebo (Joffe et al., 2006). Besides an increased and decreased inferior frontal lobule activation during storage of verbal and non-verbal material, respectively, a relatively large, randomized, double-blind, placebo-controlled, cross-over trial of estrogen treatment (for three weeks) in perimenopausal women indicated also a sharpened hemisphere encoding retrieval asymmetry (HERA) effect, with higher left-frontal hemisphere activation during encoding, and higher right-frontal hemisphere activation during retrieval in a working memory task (Shaywitz et al., 1999).

**Table 2:** **Cross-sectional studies neuroimaging cognitive functioning during HRT**.

**Study**	**Subjects [STRAW stage]**	**Age (y)**	**Hormonal treatment**	**Technique / Tracer**	**Test**	**Comparison**	**Results**	**Notes**
Berent-Spillson et al., 2012	56	(42–61)	(No HT ≥3m):	fMRI	Episodic verbal memory (encoding and storage)	PreMP vs. periMP vs. postMP	PostMP: ↑ iFC r, PFC l, TP l	Different executive function, and verbal fluency, after adj for age
	[S:−3 S:−2 S:+1]	47 ± 3	gr1 (*n* = 20): PreMP				periMP: ↑iFC l	
		53 ± 3	gr2 (*n* = 15): periMP				corr −: E and iFC l, TP l, PHC l, PC l	
		54 ± 3	gr3 (*n* = 32): PostMP					
					Visual working memory		n.s.	
Dumas et al., 2012b	24	59 ± 6	gr1 (*n* = 12): E (3m) + antimuscarinic / antinicotinic / placebo	fMRI (2x)	Visual verbal working memory + cholinergic antagonist	ET vs. placebo	[Antimuscarinic]: E: ↓ mFG l (BA10), aCG r (BA24), iPL l (BA40), Ins r (BA13), sTG l (BA22)	= Performance
	[S:+2]	60 ± 5	gr2 (*n* = 12): placebo (3m) + antimuscarinic / antinicotinic /placebo				[Antinicotinic]: E: ↑ pCun r (BA31), ↑ paracentralL (BA5), ↓ PHG r (BA34)	
Maki et al., 2011	25	60 ± 3	(HT before final menstruation):	fMRI	Verbal memory	HT vs. NT	[recognition]: NT: ↑ PHG (BA35, 36), HT: ↑HC l	HT: ↑ verbal memory performance
	[S:+2]	60 ± 3	gr1 (*n* = 13): E/CEE (P, *n* = ?)				[match]: HT: ↑ HC l	
			gr2 (*n* = 12): NT				[encoding-match]: NT: ↑ HC l	
					Figural memory	HT vs. NT	[recognition]: HT: ↑ PHG l (BA35, 27), perirhinal	
							[recognition-match]: HT: ↑ PHG r (BA35)	
Berent-Spillson et al., 2010	55	68 ± 6	PostMP (within 2y of MP and past HT≥10y):	fMRI	Visual working memory	ET+HT vs. NT	ET+HT: ↑ iFL r, aC, aIns l, PL r, sPL l, HC l, PHC, pC, raphe	= Performance
	[S:+2]	64 ± 5	gr1 (*n* = 17): CEE					Corr +: performance and activation Hipp r, meTL
		66 ± 5	gr2 (*n* = 20): CEE+P					
			gr3 (*n* = 18): NT					
						ET vs. NT	ET: ↑ sFL, aIns l, pIns, sPL r, iPL, HC l, PC	
						HT vs. NT	HT: ↑ pFC r, iFL r, sFL l, Pu l, Ins r, aIns l, pIns l, sPL r, HC, PHG l, pC, midbrain raphe	
						HT vs. ET	HT: ↑ sPC l, PHG	
							ET: ↑ sFL r, pFC r, sPC r	
Gleason et al., 2006	23	58 ± 5	PostMP	fMRI	Cognitive	HT vs. NT	HT: ↑ HC r, vpTL l	E > NT > CEE memory performance
	[S:+2]		gr1 (*n* = 4): opposed E					
			gr2 (*n* = 10): opposed CEE					
			gr3 (*n* = 9): NT					
Maki and Resnick, 2000	28	66 ± 6	gr1 (*n* = 12): E (+P, *n* = 6) (pastET≈15y) + 2y	PET (2x)	Verbal memory	ET vs. NT	ET: ↑ HC r, Ins r, sTG l	HT: ↑ memory performance
	[S:+2]	68 ± 6	gr2 (*n* = 16): NT (pastET, *n* = 5) + 2y	[^15^O] water		Verbal vs. rest	NT: ↑ ACC l	
						Over time		
					Figural memory	ET vs. NT	ET: ↑ pPHG r, iFG r	
						Figural vs. rest	NT: ↑ miTG r, ↓Cb r	
						Over time		
Resnick et al., 1998	32	68 ± 6	gr1 (*n* = 15): ET (+P, *n* = 7)	PET	Resting state	/	/	HT: ↑ figural and verbal memory
	[S:+2]	65 ± 6	gr2 (*n* = 17): NT (pastET, *n* = 5)	[^15^O] water				
					Verbal memory	ET vs. NT	ET: ↓ PHG r, PCun r, dFG r	
						Verbal vs. rest	NT: ↑ iFC r, ↓ Hy l	
					Figural memory	ET vs. NT	ET: ↑ iPL r, ↓ PHG r, NT: ↑ visual ass cortex l, ↓ aThal l, MB r	
						Figural vs. rest		

See Appendix for acronyms/abbreviations.

Moreover, two randomized controlled fMRI trials (Table 1), with cross-over designs, including ten healthy postmenopausal women each, suggested a counteractive effect of progestagens on memory, with increased, but to a lesser extent, prefrontal cortex activation after four weeks of combined estrogen-progestagen treatment vs. placebo (Smith et al., 2006; Persad et al., 2009). On the contrary, one parallel study of postmenopausal women did not observe any effect of six months of combined hormonal treatment in comparison with placebo on verbal fluency and mental rotation (Davison et al., 2013).

Results of cross-sectional neuroimaging studies on cognitive function are sparse (Table 2), and interpretation is limited by sample selection and study set-up discrepancies (Gleason et al., 2006; Berent-Spillson et al., 2010, 2012; Maki et al., 2011). Combined estrogen- or estrogen-progestagen treatment was associated with increased bilateral activation of the medial temporal lobe during encoding in postmenopausal women (Gleason et al., 2006). Activation in the medial temporal lobe during verbal and figural memory tasks differed between postmenopausal women who started HRT before menopause and never users, with a more pronounced decrease in parahippocampal gyrus and increase in left hippocampus activation in the HRT users during verbal recognition (Maki et al., 2011). Furthermore, HRT was associated with a better verbal memory performance, indicating a beneficial effect of HRT (Maki et al., 2011). A relatively large study found generalized increased brain activation upon a visual working memory task in women who had used estrogen, with or without progestagen addition, for more than 10 years, and begun HRT within two years of the menopause, compared to non-users (Berent-Spillson et al., 2010). In the same sample, activation of the medial temporal lobes correlated positively with performance during a visual working memory task (Berent-Spillson et al., 2010). Moreover, differences between pre-, peri-, and postmenopausal women during a verbal, but not visual, memory task were found, independently of age, in frontal, prefrontal, and temporal cortices, with brain region activation being negatively correlated with estrogen levels (Berent-Spillson et al., 2012).

Finally, Maki and Resnick's longitudinal PET study provided evidence that estrogen treatment, with or without progestagen addition, modulates longitudinal changes in blood flow in brain regions implicated in cognitive function. Firstly, HRT users and non-users performing a verbal memory and a visual memory task showed, in a complex fashion, differences in the activation of regions subserving memory, such as the frontal lobe (Resnick et al., 1998). A smaller and overlapping sample was investigated two years later using the same study set-up. Greater activation of the temporal gyrus, hippocampus and insula regions, and the parahippocampal gyrus and inferior frontal gyrus, was observed during verbal and visual memory tasks, respectively, in long-term HRT users (more than two years) compared to non-users, thus indicating altered blood flow in brain areas subservicing memory in the presence of HRT (Maki and Resnick, 2000). However, in non-users other regions were also activated during the figural memory task compared to users (Maki and Resnick, 2000). Moreover, HRT was associated with better memory performance in the neuropsychological tests at both time points (Resnick et al., 1998; Maki and Resnick, 2000).

Notably, different components of the cognitive tasks contribute to the complexity of the many neuropsychological functions examined (Tables 1, 2). Functional domains of memory, such as executive function, attention, visuospatial processing, and language, are subserved by different brain circuits, which may have variable sensitivity to specific interventions (Gibbs, 2010). Memory tasks have been used predominantly to assess menopausal and HRT effects on cognitive functioning. Working memory, mainly subserved by the dorsal prefrontal cortex in humans, is a limited-capacity storage system to maintain and manipulate information over short time periods and is critical for many daily activities (Gibbs, 2010). The visual-verbal N-back test of working memory, used by Dumas et al. (2010), allows the examination of different working memory loads (i.e., 0-, 1-, 2-, and 3- back conditions), from a condition of minimal memory load (0-) to one where subjects need to hold four letters in memory and update working memory as the next letter appears, alternatively seen as a cognitive stress model. Other verbal memory tasks, used by Shaywitz et al. (1999), Joffe et al. (2006), Persad et al. (2009), Berent-Spillson et al. (2012), Davison et al. (2013), involve learning and recall of word lists. Verbal memory has been suggested to be the most sensitive cognitive domain to the effect of HRT, and also a candidate proxy for dementia (Maki, 2013). The obvious discrepancy between imaging and cognition test results might be due to compensatory or deficient mechanisms, but also to differences in neuropsychological test performance within or outside the scanner. Noticeably estrogen did not seem to equally affect all cognitive domains, as shown by experimental studies (Gibbs, 2010).

fMRI detects changes in the blood oxygen level dependent (BOLD) signal, which is an index of the metabolic expenditures by neurons or glia during and immediately after the task. The BOLD signal is commonly accepted to reflect changes in neural activity, but interpretation should be cautious (Henderson and Greicius, 2010). Overall, it seems plausible to conclude that estrogen, alone or in combination with progestagens, positively affects brain activation during memory processing in postmenopausal women (Tables 1, 2). Nevertheless the neural underpinnings of HRT effects on memory remain to be elucidated. The prefrontal cortex is of particular relevance for cognition, and the neural substrate of the estradiol-induced memory enhancement, possibly due to its sensitivity to the hormonal milieu. Future studies are needed to clarify whether HRT may lower the risk of Alzheimer's disease and protect against age-related declines in memory (Henderson and Greicius, 2010; Marjoribanks et al., 2012), as well as which duration and timing of HRT would be optimal (Daniel, 2013).

#### Pharmacological menopause and cognitive functioning

Gonadotropin-releasing hormone agonist (GnRHa) treatment has been used as a model for menopause to assess cognitive signatures in young women (Table 3). GnRH is produced by the hypothalamus and pulsatile secretion of GnRH causes the anterior pituitary to release follicle-stimulating hormone (FSH) and luteinizing hormone (LH). Continuous treatment with GnRHa, on the other hand, will result in suppressed gonadal hormone production and is commonly prescribed for endometriosis, advanced breast cancer, but also to facilitate and secure subsequent safe super-ovulation during assisted reproduction therapy. GnRHa treatment results in postmenopausal levels of endogenous estradiol by the second to fourth week of administration.

**Table 3:** **Studies using GnRH agonist treatment neuroimaging cognitive functioning**.

**Study**	**Subjects [STRAW stage]**	**Age (y)**	**Hormonal treatment**	**Technique / tracer**	**Test**	**Comparison**	**Results**	**Notes**
Craig et al., 2010	30	38 ± 7	gr1: (*n* = 16) GnRH (8w) + GnRH & scopolamine	Ph-fMRI (2x)	Visual working memory + cholinergic antagonist	GnRH vs. placebo, + anticholinergic	GnRH: ↓↓ sF (BA6+9), miF (BA9+46), iF (BA45), oF (BA10), PHG l (BA34), smG gyri r (BA40), precG (BA6), iPL (BA40), aC (BA24+31)	= Accuracy performance;
	[S:−4]	40 ± 5	gr2: (*n* = 14) placebo + placebo & scopolamine					↓ response time in GnRH+scopolamine performance
		(26–47)						
Craig et al., 2009	26	39 ± 2^¤^	gr1: (*n* = 13) baseline + GnRH (8w) + GnRH & scopolamine	Ph-fMRI (3x)	Encoding and recognition memory + cholinergic antagonist	GnRH vs. placebo	GnRH: ↓iFG l (BA45)	= Encoding performance;
	[S:−4]	41 ± 1^¤^	gr2: (*n* = 13) baseline + placebo (8w) + placebo & scopolamine			GnRH vs. placebo, + anticholinergic	GnRH: ↓iFG l (BA45)	↓ recognition performance in GnRH+scopolamine
		(26–47)						
Craig et al., 2008b	13	39 ± 7	FP + GnRH (8w) + FP (after 6 m)	Ph-fMRI (3x)	Encoding and recognition memory	GnRH vs. FP preGnRH	[recognition:] ↓ IFG l	↓ Acccurancy of recognition performance during GnRH use
	[S:−4]					GnRH vs. FP post GnRH	[recognition:] ↓ IFG l	
						FP preGnRH vs. FP post GnRH	[recognition:] n.s.	
Craig et al., 2008a	34	39 ± 2^¤^	gr1: (*n* = 17) FP + GnRH (8w)	Ph-fMRI (2x)	Visual working memory	GnRH vs. placebo	[encoding:] GnRH: ↓ sPC l/paracL?(BA5), aCC l (BA24), pC l (BA31), pCun l (BA 7), PHC l (BA35), miTG l (BA21+22+39)	= Performae
	[S:−4]	41 ± 1^¤^	gr2: (*n* = 17) FP + placebo (8w)					
						GnRH vs. placebo across time	[encoding:] GnRH: ↓ Cb l/pCun l (BA7), pC l (BA31), FuG l (BA37)	
						GnRH vs. placebo	[recognition:] GnRH: ↓ Cb, BS	
						GnRH vs. placebo across time	[recognition:] Cb, FuG, iTG l	
Craig et al., 2007b	30	38 ± 2	gr1: (*n* = 15) FP + GnRH (8w)	Ph-fMRI (2x)	Verbal encoding and retrieval memory	GnRH vs. placebo	[encoding:] GnRH: ↓ mFG, iFG l (BA44, 45), aCC l, and precG r	
	[S:−4]	40 ± 1	gr2: (*n* = 15) FP + placebo (8w)			GnRH vs. placebo across time	[encoding:] GnRH: ↓ mFG, miFG, iFG l, aCC l	
		(26–47)						
Craig et al., 2007a	10	(26–47)	FP + GnRH (8w)	Ph-^1^H MRS	NAA, Cho, Cr, ml, Glx	GnRH vs baseline	[Cho, Cho/Cr:] GnRH: ↑ dlPFC l	
	[S:−4]							
Berman et al., 1997	11	35 ± 7	GnRH (8–12w)+ GnRH+E (4–5w) + GnRH+P (4–5w)	Ph-PET	Working memory	GnRH vs. GnRH+E	E: ↑ iFG, FuG l, HG l, iTL/Cb l	= Performance
	[S:−4]	(27–49)		[^15^O] water		GnRH vs. GnRH+P	P: ↑ iFG, iTG l, iPL l	
						GnRH+E vs. GnRH+P	E: ↑ HC l, mTG/iTG l	

See Appendix for acronyms/abbreviations.

Berman and colleagues performed a seminal PET study using a GnRHa-induced hypogonadism model in 1997, and found higher blood flow in the inferior frontal gyrus during estrogen- or progestagen add-back to the GnRHa treatment (Berman et al., 1997). Three subsequent fMRI studies from Craig et al., though region-of-interest based, indicated a decreased activation of the same region, i.e., the left inferior frontal gyrus, during different memory tasks, after 8 weeks of pharmacological menopause (Craig et al., 2007b, 2008a,b). Moreover, this effect was demonstrated to be reversible as reflected at re-examination six months after discontinuation of GnRHa treatment (Craig et al., 2008b). Also, restored cognitive performance has been observed after GnRHa resolution (Craig et al., 2008b), or GnRHa discontinuation plus estrogen add-back treatment (Sherwin and Tulandi, 1996).

These results essentially corroborate the neuroimaging findings on working memory (i.e., visual, encoding and recognition, verbal and retrieval memory) in postmenopausal women (Tables 1–3). It is likely that these effects are not a direct consequence of the GnRHa but rather an indirect effect of reduced estradiol and progesterone levels in the brain. However, though the pseudo menopause produced by GnRHa is a powerful model, the impact of short-term ovarian suppression on cognition in young premenopausal women seems to be contradictory; in some cases strong (Sherwin and Tulandi, 1996; Grigorova et al., 2006), and in other cases only marginal (Rocca et al., 2014) or absent (Owens et al., 2002).

Taken together, though limited by a region-of-interest analysis approach, pharmacological challenge that induced acute ovarian hormone suppression in premenopausal women leads to attenuation of the brain activation pattern in the left inferior frontal gyrus (Table 3), a region sub-serving deep processing of to-be-learned words, language and memory, typically activated during performance of executive function tasks in healthy women.

### Neurobiological functioning

#### Neurotransmitter systems

The changes in the hormonal milieu, and particularly in estrogen levels, during the menopausal transition period are likely to modulate neurotransmitter and neuroendocrine systems, thus modifying intermediate phenotypic patterns. Such neuro-psycho-biological signatures may be involved in the mechanisms by which hormone changes may elicit cognitive dysfunction (Epperson et al., 2013), mental distress, and mental disorders (Hafner et al., 1993; Hafner, 2003; Freeman et al., 2006, 2014; Riecher-Rössler and Kulkarni, 2011). Most likely, the hormonal effects on brain function highlighted by fMRI studies are indirectly mediated through hormonal actions on neural sites serving multiple neurobiological systems (e.g., cholinergic, serotonergic, noradrenergic, and dopaminergic neurotransmitter system), as indicated by studies of primates showing estrogen-mediated neurotransmitter activities in the prefrontal cortex via alteration of cholinergic and monoaminergic innervations (Voytko et al., 2009), and emphasized by the widespread distribution of estrogen and progesterone receptors in the brain (Brinton et al., 2008; Wharton et al., 2012). The cholinergic, serotonergic, and dopaminergic systems have, in fact, been preliminarily investigated in relation to menopause, and seem to be biological mediators of positive effects of estrogens (Table 4) (Henderson and Greicius, 2010).

**Table 4:** **Positron emission tomography, pharmaco-functional magnetic resonance imaging, single-photon emission computed tomography, and magnetic resonance spectroscopy neuroimaging studies of HRT**.

**Study**	**Subjects [STRAW stage]**	**Age (y)**	**Hormonal treatment**	**Technique / Tracer**	**Test**	**Comparison**	**Results**	**Notes**
Kranz et al., 2014	30	55 ± 5	gr1 (*n* = 10): E	PET (2x)	5-HT_1A_	Baseline vs. E or HT	n.s.	
	[S:≥+1B/C]	(47–64)	gr2 (*n* = 10): E+P	[carbonyl-^11^C]WAY-100635				
			gr3 (*n* = 10): placebo					
Epperson et al., 2012	8	53 ± 4	Tryptophan/sham depletion baseline + transdermal E_2_ (3–8w)	ph-fMRI (2x)	5-HT depletion + working memory	TD by ET interaction	TD [2 Back]: ↓dlPFC, miF/CG, but not after ET	= Performance
	[S:≥+1B/C]				5-HT depletion + emotion identification	TD by ET interaction	TD+ET: ↓OFC, Amy, than TD alone	= Performance
Smith et al., 2011	50	66 ± 4	HT history (within 2y of MP and ≥10y):	PET	AChE	ET vs. HT vs. NT	HT vs. NT: ↑HC l, pC	Only pC after adjustment for years of HT
	[S:+1C/2]	64 ± 5	gr1 (*n* = 13): CEE	[^11^C]PMP				
		66 ± 5	gr2 (*n* = 21): CEE+P					
			gr3 (*n* = 16): NT					
Compton et al., 2008	34	62 ± 6	gr1 (*n* = 17): E/CEE	SPET	5-HT_2A_	NT vs. ET	NT: ↑ HC	= Performance;
	[S:+1C/2]	65 ± 8	gr2 (*n* = 17): NT	^123^I-5-I-R91150				HC 5-HT_2A:_ -corr memory
Norbury et al., 2007	32	65 ± 6	gr2 (*n* = 11): MP ET (past ET +sMP)	SPET	m_1_/m_4_	ET vs. NT	ET: ↑ striatum l, HC l, lFC, Tha	↑ Performance in executive function;
	[S:+2]	65 ± 8	gr3 (*n* = 11): MP NT	(R,R)[^123^I]-I-QNB	mAChR			Corr: E and m_1_/m_4_ in TC, HC l in ET
Yue et al., 2007	182	66 ± 8	gr1 (*n* = 83): HT (>4y, !low dose)	^1^H MRS	NAA, tCR, mI	HT vs. NT	HT: ↑ NAA/tCr HC in ApoE ε 4 carriers	ApoE Genotype effect
	[S:+2]	67 ± 8	gr2 (*n* = 99): NT					
Gardiner et al., 2004	13		Baseline + CEE (4w) + CEE & P (2w)	SPET	DAT	Baseline vs. ET	ET: ↑ aPu l	
	[S:+2(?)]			[99mTc]TRODAT-1		Baseline vs. HT	HT: ↑ aPu	
Kugaya et al., 2003	10	54 ± 7	Baseline + E (10w)	PET (2x)	5-HT_2A_	Baseline vs. ET	ET: ↑ PFC r (BA9), iFG r (BA47), meFG r (BA6, 10), aCC r (BA32)	HT: ↑ verbal fluency and executive cognition performance, but not mood
	[S:≥+1B]			[^18^F]denteroaltanserin			corr +: E and 5-HT_2A_in iFG r (BA44)	
Moses-Kolko et al., 2003	5		Baseline + E (8–14w) + E and P (2–6w)	PET (3x)	5-HT_2A_	Baseline vs. ET	ET: ↑ sFG r, vlPFC r, iPL l, TL l	
	[S:≥+1(?)]			[^18^F]altanserin		Baseline vs. HT	HT: ↑ sFG, precG l, Ins l, meFG r, lOFC l, pCG r, Cun r, Tpole l, mTG, LgG l, PHG l, FuG r, meOG l	
						ET vs. HT	HT: ↑ sFG l, meOFC r, lOFC l, pCun/sPL r	
Smith et al., 2001	28	64 ± 3	(HRT within 2y MP):	SPECT	VAChT	ET vs. NT	n.s.	= Overall performance
	[S:+2]	65 ± 4	gr1 (*n* = 8): E	[^123^I]BVM		ET vs. HT	ET: ↑ pC	
		67 ± 6	gr2 (*n* = 8): E + P			yET/HT	yET/HT +corr: FC, PC, TC, aC, pC	
			gr3 (*n* = 12): NT					
Robertson et al., 2001	37	63 ± 10	[parietal lobe]:	^1^H MRS	NAA, Cr+PCr, Cho	ET vs. NT	[Cho]: NT: ↑ PL, HC	No effect of ApoE genotype
	[S:+2]	65 ± 8	gr1 (*n* = 21): E (CEE, *n* = 5; + P, *n* = 2)				[Cho]: NT: corr −: memory in HC	
			gr2 (*n* = 16): NT					
			[Hipp]:					
			gr1 (*n* = 14): E					
			gr2 (*n* = 12): NT					
Moses et al., 2000	5	52 ± 3	Baseline + E_2_ (8–14w) + E_2_ and P (2–6w)	PET (3x)	5-HT_2A_	Baseline vs. ET vs. HT	HT vs. baseline: ↑lOFC, pgACC, dlPFC, daCC, CER	Possible sole effect of E over time (DV_ROI_)
	[S:≥+1B]			[^18^F]altanserin				

See Appendix for acronyms/abbreviations.

***Acetylcholine (ACh).*** The central cholinergic system is critically involved in memory, learning and attention. Physiological aging is associated with reduction in cholinergic functional markers, such as choline acetyltransferase (ChAT), the enzyme synthetizing ACh, but with a relative preservation of cholinergic cells and terminals. Alzheimer's disease, on the other hand, is associated with drastic reductions in ChAT and diffuse degeneration of cholinergic terminals as well as loss of acetylcholine nicotinic and muscarinic receptors m1 and m4 (Gibbs, 2010; Chopra et al., 2011). Both estrogen receptors have been identified in the nuclei of the human basal forebrain, which is the source of major cholinergic innervations to the cerebral cortex, hippocampus, and hypothalamus (Gibbs, 2010). Moreover, decreased cholinergic axon density in the prefrontal cortex was found in ovariectomized monkeys compared to estrogen-only or estrogen-progesterone treated animals, as well as to control animals (Kritzer and Kohama, 1999); and cholinergic fiber density in the prefrontal, but not parietal, cortex of ovariectomized monkeys is preserved after two years of estrogen treatment (Tinkler et al., 2004). Estrogen is indeed known to provide neurotrophic support, possibly through synergistic actions with the cholinergic system, and to regulate acetylcholine release, as supported by findings in rodents and non-human primates (Voytko et al., 2009; Daniel, 2013). Thus, while low menopausal estradiol levels are likely to diminish, HRT may enhance the survival or plasticity of cholinergic cells in postmenopausal women.

A cross-sectional PET study of women who initiated HRT within 2 years of menopause, and who had used it for more than 10 years, indicated a higher cholinergic activity (8–10%), measured as acetylcholinesterase (AChE), the enzyme degrading ACh into choline and acetate, bilaterally in the posterior cingulate of combined estrogen-progestagen users compared to non-users (Smith et al., 2011). No effect on AChE was observed in the estrogen-only users compared to the non-users (Smith et al., 2011), suggesting a critical role of progesterone. On the contrary, cholinergic synaptic terminal density, assessed as binding of acethylcoline transporter (VAChT) in a SPECT cross-sectional study, was 25% higher in the parietal cortex of women treated with estrogen-only, whom initiated HRT within 2 years of menopause, compared to combined hormone users (Smith et al., 2001). Moreover, VAChT binding in frontal, temporal and parietal cortices, as well as anterior and posterior cingulate regions, was positively correlated with duration of HRT use (Smith et al., 2001), consistent with a longer exposure to gonadal steroids being associated with trophic support of cholinergic neurons. Within the cholinergic system, receptors have also been investigated using SPECT in a cross-sectional study, and higher acethylcoline muscarinic receptor m1/m4 binding was observed in postmenopausal estrogen users compared to women not receiving hormonal therapy, with greater density in the hippocampus, prefrontal cortex, striatum and thalamus (Norbury et al., 2007).

Additional proof of an interaction between estrogens and the cholinergic system has been given by two pharmaco-fMRI studies by Craig and colleagues (Table 3). This is in line with a protective effect of estrogen from the detrimental effects of anti-ACh challenge on attention found in humans (Dumas et al., 2006, 2008), monkeys (Voytko, 2002), and rodents (Sherwin and Henry, 2008). The former study demonstrated an additive effect of anticholinergic challenge with scopolamine on the decreased inferior frontal gyrus activation during encoding and recognition memory processing, in young women exposed to pharmacological menopause compared to placebo (Craig et al., 2009). The latter study, also using the cholinergic muscarinic receptor antagonist scopolamine, assessed visual working memory, and found more pronounced reduction of activation in frontal regions and parahippocampal gyrus in young women with pharmacologically induced menopause (Craig et al., 2010). Moreover, three-month estrogen treatment was shown to alter anticholinergic-related (i.e., both with the antimuscarinic drug scopolamine and with the antinicotinic drug mecamylamine) brain activation during a working memory task in postmenopausal women (Dumas et al., 2012b). Scopolamine challenge blocks muscarinic cholinergic receptors, and contributes to impairment of encoding of new memories; thus can be used as a pharmacological model of cognitive decline to investigate HRT-by-cholinergic interactive effects.

Altogether these studies reported an enhanced cholinergic neurotransmission in the presence of HRT, however discordantly regarding estrogen-only vs. estrogen-progestagens combined treatment (Tables 3, 4). The paucity and conflicting nature of findings regarding a modulatory effect of estrogen and/or progestagens on the effects of age on neural integrity in brain regions involved in cognitive function, on age-related memory decline, and on risk for Alzheimer's disease, impede any conclusion (Marjoribanks et al., 2012; Maki, 2013). Furthermore, the effect of menopausal stage at HRT initiation on cholinergic function and related hippocampus-mediated cognitive functioning needs to be explored (Daniel, 2013). For example short-, but not long-, term hormonal treatment has been shown to enhance cholinergic function in the prefrontal cortex and hippocampus of ovariectomized rats (Gibbs, 2000).

***Serotonin (5-HT).*** Effects of menopausal changes in hormonal milieu and HRT may be partly mediated via the serotonin pathway, as experimental animal models indicate effects of ovarian hormones on the serotonergic system (Bethea et al., 2009; Voytko et al., 2009; Sanchez et al., 2013). Three longitudinal, but small, PET studies assessing the serotonin receptor 2A (5-HT_2A_) highlighted a general increased HRT-mediated binding potential in frontal and cingulate regions in postmenopausal women compared to baseline (Table 4) (Moses et al., 2000; Kugaya et al., 2003; Moses-Kolko et al., 2003). Consistently, 5-HT_2A_ receptor availability in the prefrontal cortex was higher after two months of unopposed estrogen use in two independent samples of postmenopausal women (Kugaya et al., 2003; Moses-Kolko et al., 2003). Combined estrogen-progestagen treatment for one month was associated with increased 5-HT_2A_ binding potential in the frontal, precentral, cingulate, lingual, fusiform, parahippocampal and temporal gyrus, mainly in the left hemisphere, as well as in the insula, cuneus, and temporal pole in one study of five postmenopausal women (Moses-Kolko et al., 2003), and in the pregenual and dorso-anterior cingulate cortex, dorsolateral and lateral orbito prefrontal cortex, bilaterally, based on a region-of-interest approach, in another study of five postmenopausal women (Moses et al., 2000). Further support for a positive coupling between absolute levels of estradiol and global 5-HT_2A_ receptor availability comes from a large cross-sectional molecular imaging study in males (Frokjaer et al., 2010). Lastly, one SPECT study indicated lower hippocampal 5-HT_2A_ receptor binding to be coupled with estrogen treatment in long-term oophorectomized women, hypothesizing a down-regulation of post-synaptic receptors due to increased activity of the serotonergic pathway. However this result did not remain significant after correction for multiple comparison (Compton et al., 2008), and no firm conclusion can be derived from this finding due to the lack of correction for multiple comparisons and the low test-retest in the region of interest (Haugbol et al., 2007).

The mechanism by which HRT leads to a globally distributed 5HT_2A_ binding increase is not fully understood. Yet, it potentially involves hormone-mediated genomic as well as neurotrophic mechanisms. Indeed, rodent work support that 5-HT_2A_ gene transcription is affected by estradiol. In ovariectomized rats, two-week estrogen plus progesterone, however not estrogen-only, treatment was associated with increased 5-HT_2A_ mRNA in the ventral hippocampus but not in the frontal cortex (Birzniece et al., 2002). Likewise, at the protein level, 5-HT_2A_ binding in the cerebral cortex of rats was increased after two-week estrogen-only, but not combined estrogen-progesterone, treatment (Biegon et al., 1983), while in blood of depressed women was decreased after four months of hormonal treatment (Wihlback et al., 2001). Additionally, reduced 5-HT_2A_ mRNA and binding in the frontal cortex of rats was restored after a two-week estrogen treatment (Cyr et al., 1998), suggesting that hormonal withdrawal *per se* also affects 5-HT_2A_ receptor numbers. All together these findings are in line with rat studies reporting direct effects of estradiol in maintaining 5-HT_2A_ receptor levels, both after castration of male animals and after ovariectomy in female animals (Sumner and Fink, 1997, 1998; Sumner et al., 1999, 2007). Testosterone, also increase 5-HT_2A_ receptor expression, however only when converted to estradiol locally by aromatase (Sumner and Fink, 1998), again highlighting that estradiol is the main driver of 5HT_2A_ responses to HRT.

A recent randomized placebo controlled study of postmenopausal women found no effect of 8 weeks of HRT, of neither estrogen-only nor combined estrogen-progestagen treatment, on 5-HT_1A_ binding potential (Table 4) (Kranz et al., 2014). However, 5-HT_1A_ receptor binding was negatively correlated at the whole brain level with progesterone, but not estradiol, serum concentrations in postmenopausal women (Stein et al., 2014). Interestingly, an animal model of surgical menopause indicated a time-dependent effect of estrogen on this autoreceptor, with one-month, but not five-months, estrogen treatment being associated with decreased 5-HT_1A_ receptor protein expression in ovariectomized monkeys in the dorsal raphe nucleus, which is source of serotonergic neurons within CNS (Henderson and Bethea, 2008). However, combined estrogen plus progesterone administration decreased 5-HT_1A_ receptor protein expression at both time points (Henderson and Bethea, 2008). Also, 5-HT_1A_ receptor binding in the dorsal raphe nucleus has been shown to decrease after one month of treatment with either estrogen, progesterone, or a combination of both in ovariectomized monkeys (Lu and Bethea, 2002). Thus, an enhanced time-dependent effect of HRT on serotonergic neurotransmission cannot be excluded.

Additionally, using a dietary tryptophan depletion challenge, by which serotonergic tonus can be lowered transiently, serotonin-by-estrogen interaction effects were found in a small fMRI study in relation to cognitive and emotional processing in early postmenopausal women (Table 4). When tryptophan depleted, non-users had reduced bilateral dorsal lateral prefrontal cortex and middle frontal/cingulate gyrus activation in comparison to estrogen-only treated women; while lowered orbitofrontal cortex and amygdala activation in relation to emotion identification was observed in the estrogen users (Epperson et al., 2012). However, no interactive effect was observed between tryptophan or tyrosine/phenylalanine depletion and estrogen treatment on emotional, behavioral, and cognitive performance in healthy postmenopausal women (Newhouse et al., 2008, 2010). Women exhibited increased stress-triggered negative mood and worsened cognitive performance after a three-month estrogen administration compared to placebo, independently of monoamine depletion (Newhouse et al., 2008, 2010).

Estrogen, alone or combined with progesterone, not only modulates serotonergic function at the level of neurotransmitter synthesis, turnover, release, and receptor, but has also a neuroprotective role on serotonin neurons, as indicated by studies of monkeys (Bethea et al., 2009). The serotonin system critically modulates brain functions related to both cognition and mood, thus it is plausible to hypothesize a synergistic effect of estrogen and serotonin on cognitive functioning, emotion processing, and affective regulation (Bethea et al., 2009). Indeed, selective serotonin re-uptake inhibitors are valid alternatives to estradiol for treatment of vasomotor symptoms, i.e., hot flashes, and depressive symptoms (Al-Safi and Santoro, 2014; Joffe et al., 2014). Future studies, ideally translational, must elucidate how the serotonin system integrates sex-steroid hormone information. In particular, the relative contributions from increases and decreases in sex-steroid levels, pre- and postsynaptic receptor involvement, and the timing of the responses, are warranted to advance our understanding of how changes in sex-hormone milieu may trigger cognitive dysfunction, depressive symptoms and mental disorders.

***Dopamine.*** Estrogens, alone and in combination with progesterone, are likely to affect the dopaminergic system too as findings in non-human primate suggest an effect, although scattered and less prominent, of estrogen on the dopaminergic system (Voytko et al., 2009). To date one sole neuroimaging study has investigated the dopaminergic system in thirteen healthy postmenopausal women, reporting that dopamine transporter (DAT) availability in women was increased in the left anterior putamen after 4 weeks of estrogen-only treatment, and in the anterior putamen after 2 weeks of combined estrogen-progestagen treatment (Gardiner et al., 2004). Beside this study, long-term estrogen therapy has been reported to enhance dopaminergic responsivity to apomorphine in postmenopausal women (Craig et al., 2004), but further studies are needed.

#### Other neurochemical markers

MRS studies, through the detection of several metabolites (e.g., choline and N-acetyl aspartate), provide proxy measures of neural integrity of the brain, neuronal/glial membrane turnover and function in menopause (Table 4). Never-treated postmenopausal women were shown to have higher Choline (Cho)-containing compounds in the parietal lobe and hippocampus, previously identified as regions-of-interest, compared to women on HRT, and long-term visual memory was negatively correlated with Cho in the hippocampus (Robertson et al., 2001). Young women after 8 weeks of induced ovarian suppression with a GnRH agonist had higher Cho, and Cho to creatine (Cr) ratio, in the left dorsolateral prefrontal cortex (dlPFC), and borderline significant in the left hippocampus (Table 3) (Craig et al., 2007a). The Cho MRS peak mainly reflects the total amount of free acetylcholine, glycerophosphocholine, phosphocholine, and phosphoethanolamine; and Cho is a rate-limiting precursor in the synthesis of acetylcholine and a precursor of cell membrane phospholipids. These water soluble choline-containing compounds are proxy indicators of neuronal/glial cellular membrane turnover, and Cho levels are related to cognitive functioning and Alzheimer's disease (Chopra et al., 2011; Tayebati and Amenta, 2013). Increased Cho concentration may be due to loss of cholinergic neurons and/or increase in membrane phosphatidylcholine catabolism of neuronal cells to provide free Cho for the subsequent deficiency in acetylcholine production. In turn, membrane hypercatabolism may affect neuronal connectivity and signal transduction, causing impaired membrane processing (Chopra et al., 2011; Tayebati and Amenta, 2013).

Additionally, an increased N-acetyl aspartate (NAA) to total Cr (tCr) ratio in the hippocampus has been observed in postmenopausal women treated with low-dose HRT for more than 4 years compared to never-users, however, only among carriers of the dementia-related risk ε 4 allele of the apolipoprotein E (*ApoE*) gene (Table 4) (Yue et al., 2007). NAA is a neuronal marker reflecting neuronal density/function and/or mitochondrial metabolism; while tCR is mainly composed of phosphocreatine (PCr) and creatine (Cr), which are associated with high-energy phosphate metabolism, and constitute a marker for phosphate metabolism. At normal conditions tCr concentration (Cr+PCr) in the brain is very stable, which is why it can be used to normalize indexes, such as NAA/tCR. A decrease of NAA indicates damage or reduction of neurons, whereas an increase of NAA implies enhancement of the neuron metabolism thus suggesting a positive effect of HRT on neuronal function and metabolism. Nonetheless, gene-by-hormonal interactions have rarely been studied in relation to mental health in menopause. In this case, genetic risk conferred by the ε 4 allele of the *ApoE* gene (Yue et al., 2007), which has a dose-related effect on risk and onset age of AD, seems to interact with HRT, and call for independent replication in larger samples. Genetic markers associated with neurodegenerative disorders might have interactive or addictive effects on hormonal shifts effects.

All together these neurochemical changes provide initial evidence of a beneficial effect of HRT on neuronal markers (Table 4). No significant effect of HRT on the concentration of other neuro-metabolites (e.g., myo-inositol, glutamate and glutamine) quantified with MRS have so far been reported (Craig et al., 2007a; Yue et al., 2007). Myo-inositol (ml) is a putative glial marker which affects neuronal signal transduction and cellular osmolarity, and increased mI has been found in neurodegenerative diseases; while a decrease of the sum of glutamate plus glutamine (glx) has been related to loss of glutamatergic neurons, reduced synthesis or increased demand of glutamate as an amino acid.

### Brain metabolism, resting state regional blood flow and functional connectivity

Moreover, positron and single-photon computed emission tomography studies of resting state brain activity indicated a stimulating, or preserving, function of hormonal treatment for the cerebral blood flow and connectivity (Table 5), however, regions differing in cerebral glucose metabolism overlapped poorly between studies (Ohkura et al., 1995; Eberling et al., 2000; Maki and Resnick, 2000; Rasgon et al., 2001, 2005, 2014; Slopien et al., 2003; Kenna et al., 2009). Nevertheless, it remains unclear whether greater regional cerebral blood flow (rCBF) indicates better performance. PET studies assessing the rCBF employ rCBF as a marker of local neuronal activity; however questions remain about the source and meaning of the rCBF, as other mechanisms, such as changes in cerebrovascular response (vascular tone) must also be considered. SPECT also measures rCBF, and is susceptible to hemodynamic changes, since intra-cerebral vessels are responsible for the rCBF and blood-brain barrier permeability; thus increased blood flow may be attributed to effects of estrogen on the cerebrovasculature (Ohkura et al., 1995; Eberling et al., 2000; Maki and Resnick, 2000; Rasgon et al., 2001, 2005, 2014; Slopien et al., 2003; Kenna et al., 2009). Finally, two recent studies of women at high risk for Alzheimer's disease provided evidence of differential changes in brain metabolism depending on estrogen formulation and/or estrogen-progestagen HRT (Silverman et al., 2011; Rasgon et al., 2014).

**Table 5:** **Neuroimaging studies of brain metabolism HRT**.

**Study**	**Subjects [STRAW]**	**Age (y)**	**Hormonal treatment**	**Technique / Tracer**	**Test**	**Comparison**	**Results**	**Notes**
Rasgon et al., 2014	45	58 ± 4	HRT within 1y MP:	PET (2x)	rCBF	HT+ vs. HT−	HT−:↓ mPFC; iFC r, PC r	No effect of ApoE genotype
	[S:≥+1B]	58 ± 6	gr1 (*n* = 28):HT+ (E, *n* = 16; CEE, *n* = 12)	FDG		E vs. CEE	HT−(E), HT+(CEE): ↓ pCU, pCC	
			gr2 (*n* = 17): HT− (E, *n* = 13; CEE, *n* = 4)			HT+P vs. HT	HT+(E+P): ↓ PT, pCC	
							HT−(E+P): ↓ mFG	
Kenna et al., 2009	22	55 ± 3	E within 1y MP:	PET	rCBF	HT vs. NT	HT: Thal-Ln, Thal-Cn, Ln-Cn (basal ganglia) connectivity	Used as index of DA & Choline signaling
	[S:≥+1C/2]	53 ± 4	gr1 (*n* = 11): E + P (P, *n* = 9)	[^18^F]FDG				
			gr2 (*n* = 11): NT					
Rasgon et al., 2005	20	60 ± 7	gr1 (*n* = 11): E/CEE+P	PET (2x)	rCBF	Baseline HT vs. NT	n.s.	= Performance;
	[S:+2]	71 ± 9	gr2 (*n* = 9): NT	[^18^F]FDG		+2y	NT: ↓ pCC	no effect of ApoE genotype
Slopien et al., 2003	20	49 ± 5	gr1 (*n* = 20): MP baseline + E+P (1y)	SPECT	rCBF	Baseline vs HT	HT: ↑ ventricular slices (cen prefrontal reg, low pariet reg, parieto-occip reg, upp occip reg)	
	[S:≥+1B]			HMPAO				
Rasgon et al., 2001	12	65 ± 9	gr1 (*n* = 3): CEE+P; (*n* = 1): E	PET (2x)	rCBF	Baseline HT vs. NT	n.s.	No effect of ApoE genotype
	[S:≥+1C/2]	72 ± 9	gr2 (*n* = 8): NT	[^18^F]FDG		+2y HT vs. NT	ET: ↑ lateral TR	
Eberling et al., 2000	13	71 ± 8	gr1 (*n* = 8): E (≈20y)	PET	rCMRglc	ET vs. NT	?	ET: ↑ dlFC, miTG, iPL
	[S:+2]	75 ± 6	gr2 (*n* = 5): NT	[^18^F]FDG				
Maki and Resnick, 2000	28	66 ± 6	gr1 (*n* = 12): E (+P, *n* = 6) (pastET≈15y) + 2y	PET (2x)	resting state	ET vs. NT over time	ET: ↑ mi/sTG r, iTG r, miTG l	HT: ↑ memory performance
	[S:+2]	68 ± 6	gr2 (*n* = 16): NT (pastET, *n* = 5) + 2y	[^15^O] water				
Ohkura et al., 1995	14	44 ± 2	past HRT for ≥1y:	SPECT	rCBF	CEE and NT over time	ET: ↑ whole brain, Cb	
	[S:?]	43 ± 7	gr1 (*n* = 9): MP baseline + CEE (3w)	^123^I-IMP				
			gr2 (*n* = 5): MP baseline + NT (3w)					

See Appendix for acronyms/abbreviations.

Nonetheless, HRT related brain activity and connectivity responses are not well elucidated (Table 5). In particular, so far no studies of resting state MRI have been published. However, lower mPFC-hippocampal connectivity at rest has been found in healthy postmenopausal women at risk for dementia who use HRT, in the presence of high plasma insulin levels (Kenna et al., 2013). Thus, studies of reproductive aging and HRT, and their relation with brain imaging of functional connectivity are needed.

### Emotion processing

The peri menopause is often accompanied by symptoms of depression and anxiety (Al-Safi and Santoro, 2014), and studies of rodents and non-human primates have attempted to investigate the neurobiology underlying postmenopausal-like affective processing (Shively and Bethea, 2004; Mueller et al., 2014). For instance, ovariectomized female rodents displayed less anxiety-like behavior when receiving estradiol replacement (Mueller et al., 2014). In humans, increased stress-triggered negative mood after a three-month estrogen administration was noted in healthy postmenopausal women (Newhouse et al., 2008), and in older women (Dumas et al., 2012a). However, HRT has been associated with decreased depressive symptoms in menopausal women (Zweifel and O'Brien, 1997), though depending on psychiatry history, age and study design; while other studies, such as the Women's Health Initiative study, provided conflicting evidence (Henderson and Greicius, 2010). Moreover, free estradiol levels were positively correlated with mood in a recent large study of healthy women during early postmenopause (Henderson et al., 2013), but again HRT, in particular current use, was associated with symptoms of depression and anxiety in two large cohorts of peri-menopausal women (Toffol et al., 2013), however estradiol is generally recognized to have positive effects on mood (Soares, 2013).

Neuroimaging studies of emotion processing in menopausal women are essentially lacking, and findings are thus far inconclusive (Table 6). A randomized placebo-controlled cross-over study including ten postmenopausal women portrayed an increased activation, though in different regions, during negative emotional stimuli processing, in both the placebo and estrogen-progestagen treated groups, and higher activation of the left medial frontal cortex during positive stimuli processing in the placebo group (Love et al., 2010). Later, a larger cross-sectional study of long-term HRT users also showed puzzling activation in estrogen treated women and non-users, and in current vs. past HRT users (Shafir et al., 2012). While Love et al. found no effect on performance of the task (Love et al., 2010), Shafir et al. found a slower response time in HRT users, but higher accuracy in picture rating among current users compared to past users (Shafir et al., 2012). In addition, greater regional activity in the dorsal lateral prefrontal cortex during high conflict resolution was found in one study investigating emotion regulation in eleven peri- and postmenopausal women (Frey et al., 2010).

**Table 6:** **Functional magnetic resonance imaging studies of emotional processing during HRT**.

**Study**	**Subjects [STRAW stage]**	**Age (y)**	**Hormonal treatment**	**Technique**	**Test**	**Comparison**	**Results**	**Notes**
**RANDOMIZED CONTROLLED TRIAL**								
Love et al., 2010	10	57 ± 1	No HT (>3m):	fMRI	Emotion processing (positive, neutral, negative stimuli)	Placebo vs. HT	[Neg]	= Behavior
	[S:≥+1B]		gr1: E+P (4w) + wash-out (4w) and placebo (4w)				HT: ↑ OFC, OC l, precG r, pC l	
			gr2: placebo (4w) + wash-out (4w) and E+P (4w)				Placebo: ↑dlPFC l, postcG r, daC	
							[Pos]	
							Placebo: ↑ mFC l	
**CROSS-SECTIONAL STUDIES**								
Shafir et al., 2012	52	67 ± 6	HT (within 2y of MP and ≥10y):	fMRI	Emotion processing (positive, neutral, negative stimuli)	NT vs. HT	n.s.	↑ Time for picture rating in HT;
	[S:≥+2]	65 ± 5 65 ± 5	gr1 (*n* = 15): CEE			NT vs. ET	NT: Pos–Neu: ↑ mFG l (BA10), aC (BA24+32)	↑ Accuracy in current HT
							ET: Neg–Neu: ↑entorhinal cortex r	
		65 ± 5	gr2 (*n* = 20): CEE+P			NT vs. HT	NT: Pos–Neu: ↑Ins r	
			gr3 (*n* = 17): NT			Current vs. past HT	Current HT: Pos–Neu: ↑HC r	
Frey et al., 2010	11	51 ± 6	/	fMRI	Emotion regulation	High- vs. low- emotional conflict resolution	High-conflict resolution: dlPFC (BA42+40+13+9+7+Pu+30+6+Cau+30+39)	! No comparison group
	[S:≥−2]	(40–60)						

See Appendix for acronyms/abbreviations.

Monoaminergic projections throughout the prefrontal and parietal cortices are likely mediating the effect of estrogen and progestagens on emotion and mood. Cortical and limbic structures are key regulators in emotion processing; principally the dorsolateral prefrontal cortex, playing a role in the suppression of emotion, was less activated during negative emotional stimuli in estrogen-progestagen treatment compared to placebo (Love et al., 2010), and the amygdala, a key region in emotional learning, had diminished emotion-induced activation after estrogen-only treatment in the presence of dampened serotonergic function (Epperson et al., 2012).

Interestingly, in relation to the “critical window” hypothesis, are the results of a functional magnetic resonance imaging study of older women, above the age of 65 years, comparing current long-term HRT users vs. past short-term users, and fundamentally indicating no difference in emotion-induced brain activity (Pruis et al., 2009). To sum up, the precise alterations in neural activity underlying the effects of HRT on mood in menopausal women remain to be understood, since a quarter of heterogeneous neuroimaging studies is not sufficient neither to tackle the question nor to draw conclusions.

### Testosterone treatment

Testosterone treatment in postmenopausal women has been investigated by only one study. An fMRI study of nine postmenopausal women indicated a decreased activation of frontal and lingual gyri during verbal fluency, and of the parietal lobe and precuneus during mental rotation, after 6 months of transdermal testosterone treatment (Davis et al., 2013). Higher endogenous testosterone has been associated with superior performance in verbal fluency, and spatial and mathematical abilities; thus these preliminary results might indicate a more efficient brain functioning in response to cognitive tasks.

Testosterone levels are about 65% lower during menopause, and though ovarian production of testosterone continues after natural menopause, very low levels are gradually reached. Testosterone treatment is generally used for menopausal women with female sexual dysfunction, i.e., diminished sexual interest. Thus, knowledge of effect of testosterone replacement therapy in the brain should also be achieved, although it also remains to be established if putative effects are directly attributable to testosterone or rather due its conversion to estradiol, as has been showed convincingly for e.g., the 5-HT_2A_ receptor responses to HRT (Sumner and Fink, 1998).

## Discussion

Menopause is the most influential biological and health-related event for most middle-aged women. During the perimenopause (Harlow et al., 2012), which can last several years, the level of estrogen fluctuates substantially but gradually declines. The lowered estrogen levels are often accompanied by sometimes debilitating vasomotor symptoms (Harlow et al., 2012; Al-Safi and Santoro, 2014). The decision to use HRT or not represents a major concern for afflicted women and their clinicians. Effects of the menstrual cycle and pharmacological contraceptive treatment on the central nervous system, inferred from behavioral and neurophysiological activity changes in response to cognitive and emotional stimuli, indicate a role of estrogen and progesterone on brain function in healthy fertile women (Mueller et al., 2014; Toffoletto et al., 2014). Alike, menopausal hormone changes are likely to modulate neuronal activity and contribute to age-related memory loss as well as the development of disorders such as dementia or major depression (Tables 1–6).

It is tempting to conclude that the present findings reflect true alterations in neural activity in the face of a hormonal perturbation (Tables 1–6). However, the interpretation of the results presented in this review remains a matter of debate, since less activation during task performance can be interpreted either as a more efficient use of neuronal resources or as an impaired state in which adequate activation of resources cannot be executed. Vice versa, higher activation can be interpreted as beneficial, or imply a compensatory response, or a less efficient use of resources (Henderson and Greicius, 2010). Interestingly, most of the effects were observed after a relatively brief time of HRT use. Thus, the optimal duration, or dose of HRT, or timing of treatment start, necessary to maintain a premenopausal cerebral state or to trigger neuroplasticity in mature women, is unknown.

Peri- and postmenopause are marked not only by vasomotor symptoms, but also by cognitive and emotional complaints that affect quality of life and overall functioning; however the degree to which the decline in estrogen and/or progesterone production is responsible is not yet clear. Increasing evidence from observational and HRT studies of postmenopausal women supports the influence of ovarian hormones on cognition and mood both at the behavioral and neural level, although it remains ambiguous whether this influence is positive or harmful (Henderson and Greicius, 2010). In the reviewed studies, estrogen altered the activation of brain structures underlying memory performance, particularly the fronto-cingulate region; however there appears to be a puzzling dissociation between neuropsychological performance and neural activity, since many studies that found a hormonal-dependent effect in neuroimaging data did not observe neuropsychological alterations across hormonal conditions (Tables 1–3). Also, cerebral activity changes are task-dependent, as shown by experimental models (Sherwin and Henry, 2008; Gibbs, 2010), and may as well be biased by differences in education and health of the women. Longitudinal observations are hence indispensable to identify if, and at which critical age, the metabolic decline may become appreciable (Henderson and Greicius, 2010). Thus, abnormal activation when associated with normal task performance may reflect compensatory cerebral metabolic activity in response to a subtle/subclinical loss of cognitive capacity, and precede cognitive impairment in individuals at increased risk for neurodegenerative and psychiatric disorders. However, an alternative explanation to group differences in activation patterns during memory processes may be that postmenopausal women activate a distinct cognitive network. Overall, it seems plausible to conclude that estrogen, alone or in combination with progestagens, positively affects brain activation during memory processing in postmenopausal women (Tables 1, 2), likely via interactions with basal forebrain cholinergic projections (Gibbs, 2010). This suggestion is also in line with findings of preserved visual memory in ovariectomized monkeys treated with estrogen, alone or in combination with progesterone, compared to placebo-treated ovariectomized monkeys (Voytko et al., 2009). In contrast, results regarding cerebral functional responsiveness to emotional stimulation are lacking and far from being consistent (Table 6).

Furthermore, studies of neurochemical pathways have pin-pointed the cholinergic, serotonergic, and dopaminergic systems as biological mediators of estrogen influences on the brain (Table 4). Cholinergic and monoaminergic wide-spread projections to prefrontal cortical regions interactively mediate several functions ranging from working memory to emotion processing. For instance, quantitative and qualitative differences in density of axons immunoreactive to ChAT, dopamine ß-hydroxylase, tyrosine hydroxylase and serotonin in the dorsal medial prefrontal cortex was found in ovariectomized monkeys compared to control animals, and following 1 month treatment with estrogen-only or estrogen-progesterone (Kritzer and Kohama, 1998, 1999). The interplay between these neurotransmitter systems is of relevance for schizophrenia and depression, two mental disorders characterized by sex differences in their occurrence, and their interaction with hormonal changes is likely to impact treatment response.

More, knowing that cerebral blood flow decreases with age, positron, and single-photon computed emission tomography studies suggested a preservative/stimulating function of estrogen for the cerebral blood flow (Table 5). In view of these findings (Tables 1–5), and of studies of monkeys (Voytko et al., 2009), a putative neuroprotective role of estrogen in neuronal integrity maintenance and cognitive function preservation against regional cerebral metabolic decline could thus be anticipated/predicted, also considering meta-analytical findings reporting an effect of about one-third reduction in the risk of Alzheimer's disease among HRT users (Yaffe et al., 1998; Hogervorst et al., 2000; LeBlanc et al., 2001; Nelson et al., 2002). However, as highlighted by the Women's Health Initiative project, timing is likely to be critical for positive HRT effects on cognition (Henderson and Greicius, 2010).

Of special interest in relation to neurogenesis and synaptic plasticity is the hippocampus, a crucial region in memory and learning (Gibbs, 2010). Preclinical research has highlighted several effects of estrogen on structure and function of the hippocampus in animal experiments, also via effects on cholinergic projections from the forebrain, ranging from neurotrophic to neuroplastic to neurobehavioral effects (Voytko et al., 2009; Wnuk et al., 2012; Daniel, 2013). The hippocampus is atrophied and functionally impaired in Alzheimer's disease and in the presence of a history of repeated depressive episodes, neurobiological features which are linked to cognitive function. Larger hippocampal volume in HRT users has been indicated by magnetic resonance imaging studies, with the first evidence reported by Eberling et al. (2003). Several experimental and clinical studies have in fact provided evidence of a positive modulatory effect of estrogen use on hippocampal anatomy, though aversive or absent effects have also been reported (Erickson et al., 2005, 2010; Boccardi et al., 2006; Lord et al., 2008; Espeland et al., 2009; Wnuk et al., 2012). Discordance of the findings and lack of longitudinal randomized controlled trials leaves room for equivocal conclusions on the effects of HRT on hippocampus morphology. However, several neuroimaging studies indicated an enhancing effect of HRT on hippocampus function (Maki and Resnick, 2000; Robertson et al., 2001; Gleason et al., 2006; Norbury et al., 2007; Berent-Spillson et al., 2010; Maki et al., 2011; Smith et al., 2011; Shafir et al., 2012), suggesting potential positive estrogen-mediated effects on neuronal survival and plasticity through the cholinergic system (Daniel, 2013). Thus hippocampal, together with prefrontal cortical, function is a strong candidate substrate mediating the hormonal effects on cognition.

Estrogens interact with neuronal networks at many levels and affect brain development and aging (Sherwin and Henry, 2008). The modulation by estrogen may therefore account for hormonal regulation of cognition and mood, as for instance women have increased risk for developing Alzheimer's dementia and major depression compared to men, but the pathophysiological mechanisms behind these differences are unknown (Henderson and Greicius, 2010). Non-invasive measurement of brain function through neuroimaging techniques is advantageous compared to traditional behavioral measures. The BOLD signal and the rCBF are proxy outcomes, endpoints which can be objectively measured and are potentially related to a clinical phenotype, such as working memory (Henderson and Greicius, 2010). Biological relevance could thus be inferred by the effect of HRT on neuronal activity; consequently the question whether certain intermediate phenotypes can be classified as endophenotypes (Lenzenweger, 2013) for neurodegenerative and psychiatric disorders occurring during menopause remain to be addressed. Neuroimaging genetics of the peri- and postmenopause and HRT use is required to provide knowledge about constitutional vulnerability to hormonal changes in relation to mental health (e.g., the first neuroimaging genetic study of premenstrual dysphoric disorder, Comasco et al., 2014), as well as molecular research will be essential to elucidate epi-genotype-mediated mechanisms. To date there is a lack of focus on genetic correlates of menopausal hormonal effects, with only four studies having considered the *ApoE* genotype (Rasgon et al., 2001, 2005; Robertson et al., 2001; Yue et al., 2007). For instance polymorphisms in the estrogen receptor alpha and beta genes (*ESR1* and *ESR2*) have been associated with cognitive impairment in old women in two large longitudinal studies (Yaffe et al., 2002, 2009), but their potential intermediate neuronal correlates have not been investigated. Additionally, the modulatory association between the catecholamine degrading enzyme catechol-O-methyltransferase (*COMT*) and dorso and ventro lateral prefrontal cortex and supragenual anterior cingulate cortex activation during working memory has been demonstrated in schizophrenia (Rasetti and Weinberger, 2011), but remains to be explored in relation to menopausal cognitive decline.

Future studies will thus need to investigate whether putative intermediate phenotypes are involved in the causal pathway between genes and psychopathology, besides having a mere associative or consequential relationship with the illness, as well as the heritability of the endophenotype(s) (Lenzenweger, 2013). By means of such approach, differences between statistical vs. biological significance can be further discerned.

Widespread classical nuclear (i.e., estrogen receptor α and β, progesterone receptor A and B), and non-classical membrane-associated (e.g., G protein-coupled estrogen receptors and the progesterone receptor membrane component 1), receptors are targeted by estradiol and progesterone, thus mediating their effects on brain structure as well as cognitive-affective processing (Brinton et al., 2008; Wharton et al., 2012). Remarkably, estrogen replacement treatment has been the most studied (Tables 1–6), even though it is far less often used compared to combined estrogen-progestagen treatment. Estrogen-only treatment is prescribed only for hysterectomized women, as the progestagen addition is needed for protection against endometrial hyperplasia and cancer. Moreover, two recent studies of women at high risk for Alzheimer's disease provided evidence of differential changes in brain metabolism depending on estrogen formulation and/or estrogen-progestagen HRT (Silverman et al., 2011; Rasgon et al., 2014). Lately, a study found a positive correlation between serum progesterone, but not estrogen, levels and verbal memory and global cognition in healthy women during the early postmenopause (Henderson et al., 2013). Thus, potential cumulative neuroprotective or counteracting effects of progestagens—via its metabolites or indirectly via type-A γ-aminobutyric acid receptor signaling modulation—still need to be elucidated.

Importantly sampling differences between the studies also need to be considered since a gynecological clinic-based sample (e.g., women who underwent oophorectomy with or without hysterectomy, i.e., surgical menopause) is likely to differ from a sample of asymptomatic healthy menopausal women (Rocca et al., 2014). Furthermore, natural menopause is typified by a gradual decline of estradiol and androgen levels until circulating levels are exclusively maintained by peripheral conversion of adrenal steroids (Harlow et al., 2012; Al-Safi and Santoro, 2014). On the contrary, surgical menopause causes a drastic and complete cessation of both estradiol and testosterone secretion. Finally, efforts to stage reproductive aging according to standardized nomenclature and classifications, as the Stages of Reproductive Aging Workshop (STRAW) criteria, should be made to facilitate comparisons between studies (Harlow et al., 2012).

Hormonal supplementation in peri- and postmenopausal women remains a source of great health concern. The so-called “window of opportunity” or “critical period” hypothesis cannot be rejected based on the present results, but it is expectable that timing of HRT (i.e., age and proximity to menopause when HRT is initiated) will imply a different brain response, and that chronological and reproductive aging matter too (Henderson and Greicius, 2010; Daniel, 2013; Maki, 2013; Rocca et al., 2014). A critical threshold below which the function of neurobiological systems remains impaired by loss of ovarian function and cannot be triggered by exogenous hormonal supplementation is indeed plausible.

## Conclusions

Questions to-be-answered:

- What are the neurobiological mechanisms underlying menopause hormonal transition and HRT effects in the brain?
- Are there synergistic or counteracting effects of estrogen together with progestagens and/or androgens?
- Which dosage and combination of HRT should be prescribed?
- If and when should HRT be initialized and terminated to be most efficient in optimizing women's mental health?

Collectively, neuroimaging studies indicate altered brain activation patterns by estrogen-only or estrogen-progestagen treatment in therapeutic doses in postmenopausal women, corroborating preclinical findings of effects of estrogen on neural structure and function in mature animals. These results call for further well-powered randomized-controlled multi-modal neuroimaging studies, using a battery of standardized neuropsychological tests, as well as for complementary investigation on the correlated molecular mechanisms of reproductive aging and HRT in preclinical models.

## Key concepts

- Menopausal transition: period in a women's life lasting several years around the age of fifty where ovarian hormone levels, estrogens and androgens, fluctuate and decrease until menopause occurs with the termination of the menstrual cycle, and post-menopause is established; this physiological transitional hormonal change (peri-menopause), as well as surgically induced menopausal transition, are of relevance to the aging female brain.
- Hormonal replacement therapy (HRT): exogenous supplementation of estrogen and/or progesterone given during peri-menopause to treat vasomotor symptoms. The potential protective and/or aversive effects, and the biological mechanism of action of HRT, as well as the effects of interrupting HRT, on cognitive and emotional functioning remain largely elusive.
- Window of opportunity: crucial period of time spanning from peri- to post-menopause during which HRT could be used with beneficial effects on women's physical and mental health.
- GnRHa hormone manipulation: treatment with a gonadotropin releasing hormone agonist that initiates a biphasic response, which initially stimulates ovarian sex-steroid hormone production and subsequently induces a pharmacological pseudo-menopausal state, which is established by the second to fourth week of administration, with a complete cessation of both estradiol and testosterone secretion. It provides a model of early menopause and menopausal transition.

## Author contributions

Erika Comasco, Inger Sundström-Poromaa, and Vibe G. Frokjaer: study conception; literature acquisition; data interpretation; drafting the work; revising the work critically for important intellectual content; final approval of the version to be published; and agreement to be accountable for all aspects of the work in ensuring that questions related to the accuracy or integrity of any part of the work are appropriately investigated and resolved.

### Conflict of interest statement

The authors declare that the research was conducted in the absence of any commercial or financial relationships that could be construed as a potential conflict of interest.

## Appendix

### Acronyms/abbreviations in the tables

aC: anterior cingulate; aCC: anterior cingulate cortex; AChE: acetylcholinesterase; BS: brain stem; Cau: caudate; Cb: cerebellum; CEE: conjugated equine estrogen; CER: cerebellar cortex; CG: cingulate gyrus; Cn: caudate nucleus; Cun: cuneus; daCC: dorsal anterior cingulate cortex; dlFC: dorsolateral frontal cortex; dFG: dorsal frontal gyrus; dlPFC: dorsolateral prefrontal cortex; EC: entorhinal cortex; E: estrogen; FC: frontal cortex; FDG: fluorodeoxyglucose; FG: frontal gyrus; FL: frontal lobe; FP: follicular phase; FuG: fusiform gyrus; gr: group; HC: hippocampus; HERA: hemisphere encoding retrieval assimetry; HG: hippocampal gyrus; HMPAO: hexamethylpropylene-amino oxime; HT: hormonal treatment (E+P); Hy: hypothalamus; iF: inferior frontal; iFC: inferior frontal cortex; iFG: inferior frontal gyrus; Ins: insula; ipFG; inferior posterior frontal gyrus; iPL: inferior parietal lobe; iTG: inferior temporal gyrus; iTL: inferior temporal lobe; LgG: lingual gyrus; Ln: lentiform nucleus; lOFC: lateral orbito frontal cortex; mAChR: muscarinic acetylcholine receptors; MB: mammillary body; MF: medial frontal; mesial-TR: mesial temporal region; mFC: medical frontal cortex; meFG: medial frontal gyrus; meOFC: medial orbitofrontal cortex; meOG: medial occipital gyrus; miF: middle frontal; miFG: middle frontal gyrus; mFL: medial frontal lobe; miTG: middle temporal gyrus; MP: menopause; n: number of individuals; Neg: negative pictures; Neu: neutral pictures; NT: no treatment; OC: occipital cortex; oF: orbital frontal; OFC: orbitofrontal cortex; OL: occipital lobe; P: progesterone/progestins; pC: posterior cingulate; PC: parietal cortex; pCC: posterior cingulate cortex; PCL: paracentral lobule; pgG: postcentral gyrus; pCun: precuneus; paracL: paracentral lobule; PHG: parahippocampal gyrus; PL: parietal lobe; pgACC: pregenual anterior cingulate cortex; pIns: posterior insula; pCG: posterior cingulate gyrus; PFC: prefrontal cortex; PL: parietal lobe; Pos: positive pictures; post-cG: postcentral gyrus; pPHG: posterior para-hippocampal gyrus; pre-cG: precentral gyrus; Pu: putamen; rCMRglc: regional cerebral glucose metabolism; sF: superior frontal; sFG: superior frontal gyrus; smG: supra-marginal gyri; sPC: superior parietal cortex; sTG: superior temporal gyrus; STRAW: Stages of Reproductive Aging Workshop [ranging from −5 to +2] (Harlow et al., 2012); TC: temporal cortex; TD: tryptophan depletion; thal: thalamus; TL: temporal lobe; TP: temporal pole; TR: temporal region; VAChT: vesicular acetylcholine transporter; vlPFC: ventrolateral prefrontal cortex; vpTL: ventral posterior temporal lobe.

^¤^SEM: standard error of the mean.
